# Dopaminergic Medication Modulates Learning from Feedback and Error-Related Negativity in Parkinson’s Disease: A Pilot Study

**DOI:** 10.3389/fnbeh.2016.00205

**Published:** 2016-10-24

**Authors:** Chiara Volpato, Sami Schiff, Silvia Facchini, Stefano Silvoni, Marianna Cavinato, Francesco Piccione, Angelo Antonini, Niels Birbaumer

**Affiliations:** ^1^Department of Behavioural Neuroscience, IRCCS Fondazione Ospedale San CamilloVenice, Italy; ^2^Department of Medicine—DIMED, University of PaduaPadua, Italy; ^3^Parkinson and Movement Disorders Unit, IRCCS Fondazione Ospedale San CamilloVenice, Italy; ^4^Institute for Medical Psychology and Behavioural Neurobiology, University of TübingenTübingen, Germany

**Keywords:** reward learning, dopamine agonists, levodopa, error-related negativity, Parkinson’s disease

## Abstract

Dopamine systems mediate key aspects of reward learning. Parkinson’s disease (PD) represents a valuable model to study reward mechanisms because both the disease process and the anti-Parkinson medications influence dopamine neurotransmission. The aim of this pilot study was to investigate whether the level of levodopa differently modulates learning from positive and negative feedback and its electrophysiological correlate, the error related negativity (ERN), in PD. Ten PD patients and ten healthy participants performed a two-stage reinforcement learning task. In the Learning Phase, they had to learn the correct stimulus within a stimulus pair on the basis of a probabilistic positive or negative feedback. Three sets of stimulus pairs were used. In the Testing Phase, the participants were tested with novel combinations of the stimuli previously experienced to evaluate whether they learned more from positive or negative feedback. PD patients performed the task both ON- and OFF-levodopa in two separate sessions while they remained on stable therapy with dopamine agonists. The electroencephalogram (EEG) was recorded during the task. PD patients were less accurate in negative than positive learning both OFF- and ON-levodopa. In the OFF-levodopa state they were less accurate than controls in negative learning. PD patients had a smaller ERN amplitude OFF- than ON-levodopa only in negative learning. In the OFF-levodopa state they had a smaller ERN amplitude than controls in negative learning. We hypothesize that high tonic dopaminergic stimulation due to the dopamine agonist medication, combined to the low level of phasic dopamine due to the OFF-levodopa state, could prevent phasic “dopamine dips” indicated by the ERN needed for learning from negative feedback.

## Introduction

The dopamine system plays a crucial role in reinforcement learning. In particular, electrophysiological studies in primates have shown that reward elicits phasic dopamine increases, whereas aversive feedback leads to phasic dopamine decreases in midbrain dopamine neurons. Thus, reduction of phasic dopamine responses, due to a decrease or an excessive increase of dopamine level, could negatively affect prediction error and, consequently, impair reinforcement learning (Schultz, 2002; Montague et al., 2004).

Parkinson’s disease (PD) is a valuable model to study reinforcement learning because of the dysfunction involving dopaminergic basal ganglia-prefrontal cortex circuits. In PD loss of dopamine determines modification of both tonic and phasic aspects of dopamine neurotransmission affecting reinforcement learning (Frank et al., 2004). Dopaminergic medication has been reported to produce positive effects on some cognitive impairments (i.e., cognitive flexibility) and negative effects on others, such as reward learning (Swainson et al., 2000; Cools et al., 2001, 2003, 2006; Bódi et al., 2009; Peterson et al., 2009; Shiner et al., 2012). According to Frank (2005), in PD two different neural populations in the striatum respond in opposite directions to dopaminergic medication. Increased levels of dopamine activate the direct “Go” pathway (via D1 excitatory receptors) improving learning from positive feedback and suppress the indirect “No-Go” pathway (via D2 inhibitory receptors) affecting learning from negative feedback. More recently, Moustafa et al. (2013) proposed a reinforcement learning model that takes into account the different effects of levodopa and dopamine agonists on tonic and phasic dopamine levels, determining selective effects on reward learning processes. The model assumes that levodopa acts on both D1 and D2 dopamine receptors whereas dopamine agonists have a high affinity for D2 receptors. Moreover, phasic dopamine activates D1 receptors, whereas tonic dopamine activates D2 receptors only. Thus, both levodopa and dopamine agonists restore tonic activation whereas only levodopa, but not dopamine agonists, restores phasic activity. The model, therefore, predicts that levodopa and dopamine agonists could differentially affect reward learning. Levodopa enhances reward learning via phasic dopamine activity whereas dopamine agonists impair reward learning by increasing tonic dopamine activity, preventing phasic responses from being effective. These assumptions are substantiated by studies on healthy people reporting that administration of dopamine agonists negatively affects reinforcement-based learning (Frank and O’Reilly, 2006; Santesso et al., 2009).

The error related negativity (ERN) could have a peculiar function in reinforcement learning. A prevailing hypothesis holds that the ERN reflects midbrain dopamine system activity caused by dips in dopamine 50–100 ms after incorrect responses. The midbrain dopamine system conveys this error signal to the anterior cingulate cortex, where the signal is used to improve task performance (Falkenstein et al., 1991; Gehring et al., 1993; Holroyd and Coles, 2002). Phasic dopaminergic activity seems to be particularly sensitive to the alteration of dopamine levels affecting learning mechanism. In PD the alteration of tonic and phasic dopamine activity could affect ERN (Falkenstein et al., 2001; Holroyd et al., 2002; Beste et al., 2009) and learning processes (Frank et al., 2004). On the other hand, dopaminergic medication, in particular dopamine agonists, could determine a negative effect on learning. Indeed, high tonic activity of dopamine due to dopamine agonists could disturb the equilibrium of phasic activity (such as “dopamine dips” associated with the ERN) needed for reinforcement learning effects (Cools et al., 2001). Nevertheless, previous studies in PD patients showed that the ERN is not affected by dopaminergic treatment (Stemmer et al., 2007; Willemssen et al., 2008).

According to the model of Frank et al. (2005), as the difference in positive/negative feedback learning depends on different levels of dopamine in the basal ganglia, the ERN should be larger for low levels than high levels of dopamine. Dopaminergic medication could excessively increase the level of dopamine by preventing “dopamine dips” (ERN) associated with the error detection determining a specific impairment in learning from negative feedback. In their model, however, they refer to a general effect of dopaminergic medication and do not distinguish the possible differential effect of levodopa and dopamine agonists. On the other hand, according the model of Moustafa et al. (2013), only levodopa, but not dopamine agonists, should affect phasic dopamine activity, including “dopamine dips” resulting in the ERN.

In this pilot study, learning from feedback and the ERN were assessed in a small sample of PD patients performing a probabilistic learning task (Frank et al., 2004, 2005). As first objective we were interested to investigate the effect of phasic dopamine release related to levodopa bioavailability on reinforcement learning. Thus we tested PD patients in two different conditions: (1) ON-levodopa (within 1 h of levodopa therapy); and (2) OFF-levodopa (within 30 min before the next levodopa dose). From an ethical point of view, this type of measurement of the OFF-state avoided PD patients discomfort determined by forced full wash out from medication, and in our specific case, allowed patients to continue their rehabilitation program. Moreover, the stable level of dopamine agonists (all extended release formulations) resulting in tonic dopaminergic stimulation, allows to isolate the assumed (phasic) effect of levodopa on reinforcement learning without concomitant interference from the consequence of additional tonic level changes (Moustafa et al., 2013). Thus, we could expect that reward learning is facilitated in the ON-levodopa state and it is impaired in the OFF-levodopa state. In the same way, the ERN amplitude should be reduced in the OFF-levodopa state, because the ERN is affected by the lack of phasic bursts of dopamine neurons and loses the capacity to indicate violation of positive and negative expectations (Schultz, 2002; Moustafa et al., 2013). However, a further study in which both the treatment with dopamine agonists and levodopa are manipulated separately is needed to discern the specific effect of one or the other substance.

## Materials and Methods

### Participants

Ten PD patients (recruited at San Camillo Hospital of Venice) and 10 healthy participants matched for age and education were enrolled in the study. PD was diagnosed according to the United Kingdom PD Society brain bank diagnostic criteria for PD (Hughes et al., 1992); symptoms were evaluated with the Unified PD Rating Scale (Fahn and Elston, 1987) when patients were in OFF-levodopa state, and PD was staged according to the Hoehn-Yahr Scale (Hoehn and Yahr, 1967). Exclusion criteria were: (1) other neurological or mental disorder except for mild depressive symptoms (Beck Depression Inventory score (BDI) >13 (Beck et al., 1961); (2) Mini Mental State Examination (MMSE) score <24 (Magni et al., 1996); (3) advanced symptoms (stage IV or V in the Hoehn-Yahr Scale); (4) deep brain stimulation implant or continuous infusion pump. All patients took levodopa and nine took also dopamine agonists (extended release formulations) and they were stable on their medication dose for at least 2 months (Table 1). The study was conducted in accordance with the Declaration of Helsinki and was approved by the local Research Ethics Committee.

**Table 1:** **Demographic and clinical data of sample**.

Parameters	Parkinson’s disease patients	Healthy controls
Gender (m/f)	7/3	4/6
Age (y)	56.3 (3.24)	57.9 (7.5)
Education (y)	12.0 (4.24)	13.1 (4.14)
Disease duration (y)	8.6 (3.8)	-
Unified Parkinson’s Disease Rating Scale III	26.8 (15.81)	-
Hoehn-Yahr Scale	1.95 (0.15)	-
Levodopa equivalent dose (mg)	792.5 (450.0)	-
Dopamine agonists equivalent dose (mg)	198.1 (145.4)	-
Mini Mental State Examination	29.2 (1.0)	28.9 (1.37)
Beck Depression Inventory	8.8 (4.8)	9.7 (4.23)

*Table represents mean (standard deviation) of clinical and demographic data of Parkinson’s disease patients and healthy controls*.

### Procedures

The PD patients were tested in two experimental conditions separated by a minimum of 7 days: (1) within 1 h of levodopa therapy, when they experienced the maximum therapeutic effect of medication (ON-levodopa) and (2) within 30 min before the next levodopa dose, when they experienced the minimum therapeutic effect of medication (OFF- levodopa; Nombela et al., 2012).

### Task

We used a probabilistic learning task consisting of a learning phase and a testing phase (Frank et al., 2004, 2005). The stimuli were pairs of colored pictures representing common objects (International Affective Picture System; Lang et al., 2008). The stimuli were delivered by a software for experimental psychology designs (E-prime).

In the Learning Phase, three different stimulus pairs (AB, CD, EF) were presented in random order on the screen of a computer. The stimuli remained on the screen for 2500 ms. A fixation point was presented during the inter-trial interval (250–750 ms). Participants were required to identify the correct object in the pair of stimuli by pressing the key on the left side keyboard (Z) when they chose the stimulus on the left of the screen, or the key on the right side of the keyboard (M) when they chose the stimulus on the right of the screen. A probabilistic visual feedback followed the choice to indicate whether it was correct or incorrect: a green “V” for a correct response and a red “X” for an incorrect response. In the AB pair, stimulus A was followed by a positive feedback in 80% and by a negative feedback in 20% of the trials, whereas stimulus B was followed by a negative feedback in 80% and by a positive feedback in 20% of trials. In CD pairs, stimulus C was followed by a positive feedback in 70% of the trials and by a negative feedback in 30% of the trials, whereas stimulus D was followed by a negative feedback in 70% of the trials and a positive feedback in 30% of the trials. In the EF pair, stimulus E was followed by a positive feedback in 60% of the trials and by a negative feedback in 40% of the trials whereas stimulus F was followed by a negative feedback in 60% of the trials and by a positive feedback in 40% of the trials. When the participants exceeded the stimulus presentation time limit, the message “too late!” appeared on the screen and the trial was excluded from the analysis. Three blocks of 60 stimuli were performed. The trials were presented in a randomized order. During the Learning Phase, participants learned to choose stimuli A, C and E, which were followed more frequently by a positive feedback and to avoid stimuli B, D and F, which were followed more frequently by a negative feedback. It was also explained that the correct identification of the stimulus did not follow any logic beyond the feedback. To familiarize participants with the task, they were engaged in a preliminary practice block.

Immediately after, the Testing Phase followed to verify whether participants learned more from positive vs. negative feedback. For this purpose participants were tested with novel combinations of stimuli in a random sequence (AC, AD, AE, AF, BC, BD, BE, BF) in addition to the same stimulus pairs used in the Learning Phase. In the novel combinations, the stimulus that was more frequently followed by a positive feedback (A) in the Learning Phase and the stimulus that was more frequently followed by a negative feedback (B) in the Learning Phase were paired to one of the other stimuli (C, D, E, F). According to the principle of generalization of learning, in the novel combinations participants should tend to choose stimulus A and to avoid stimulus B. Accuracy was defined as the choice of A in AC, AD, AF, AE, AF pairs and the avoidance of B in BC, BD, BE, BF pairs. The trials were presented in a randomized order. Patients were instructed to follow their instinct in choosing the correct stimulus, since in the Testing Phase they did not receive any feedback. As in the Learning Phase, the participants were under time pressure. They had to respond within 2500 ms of stimulus presentation time and if they exceeded that time limit, the message “too late!” appeared on the screen. Also in the Testing Phase, participants performed a short practice block to familiarize themselves with the task. Accuracy was defined as the choice of A and the avoidance of B.

Two parallel versions of the task including different stimuli were administered during ON- and OFF-levodopa medication to avoid a repetition learning effect. The two versions of the task were counterbalanced between the ON- and OFF-levodopa condition. Moreover, the order of the ON- and OFF-levodopa condition was counterbalanced across patients.

### Event-Related Potential Recording and Analysis

During task performance, the electroencephalogram (EEG) was recorded from a 29-electrode cap with a standard 10–10 position connected to an amplifier (Neuroscan, Sterling, VA, USA). The EEG signal was referenced to Fpz. The vertical and horizontal electrooculograms were recorded from electrodes placed below the right eye and on the outer canthi of the right eye. Electrode impedances were kept below 10 kO. The EEG data were sampled at 500 Hz, amplified and filtered through a bandpass of 0.1–70 Hz. The EEG signal was processed using a MATLAB toolbox (EEGLAB v10.2.5.8b, Delorme and Makeig, 2004). The signal was low-pass filtered at 30 Hz. Ocular artifacts were removed using a MATLAB toolbox (Gratton et al., 1983). Response-locked event-related potentials were computed within epochs starting 800 ms prior to the response and lasting 500 ms after the response and were baseline corrected with respect to the first 100 ms of these epochs. ERN was defined as the peak-to-peak voltage difference between the negative peak within 150 ms after the response and the preceding positive peak (Falkenstein et al., 1991; Gehring et al., 1993). Analyses focused on signals from the Cz electrode where the ERN was easily detectable. Epochs were extracted and averaged for the following conditions: errors in positive learning items (avoidance of the A stimulus in AC, AD, AE, AF pairs) and errors in negative learning items (choice of the B stimulus in the BC, BD, BE, BF pairs) and for each group: controls, PD OFF-levodopa and PD ON-levodopa.

### Data Analysis

Since we were interested in investigating whether dopaminergic medication differently affects learning from positive vs. negative feedback in PD, we focused the analysis on the accuracy of novel pair combinations in the Testing Phase and the ERN associated to errors committed in these trials. Novel pair combinations provide the amount of learning from positive feedback (number of times the subjects chose the stimulus A) vs. negative feedback (number of times the subject avoided the stimulus B). Moreover, we analyzed the event-related potential associated to the detection of avoidance of A and the choice of B errors in the Testing Phase: the ERN. Indeed, as reported by Frank et al. (2005) in their study on healthy subjects the size of the ERN could express the degree to which participants learn more from the negative vs. the positive consequence of their decisions (feedback). Statistical analysis was performed with the IBM SPSS Statistics software package. Kolmogorov-Smirnov tests confirmed the assumptions of normality of all the variables; thus we decided to use parametric testing. Overall accuracy and overall ERN within the PD group (ON vs. OFF) was compared with a Paired Sample *T* test. A repeated measures analysis of variance (ANOVA) with two within factors “levodopa state” (ON vs. OFF) and “learning” (positive vs. negative) was applied to compare accuracy and ERN associated to positive or negative feedback within the PD group ON- and OFF-levodopa. The comparison of overall accuracy and overall ERN between groups (CTRL vs. PD) was performed with an Independent Sample *T* test. A repeated measures ANOVA with a within factor “learning” (positive vs. negative) and a between factor “group” (controls vs. PD ON-levodopa; controls vs. PD-OFF levodopa) was performed to compare accuracy and ERN associated to positive vs. negative feedback. Multiple comparisons were performed with Bonferroni correction.

## Results

### Demographic and Clinical Data

No statistically significant differences were found between PD patients and controls with respect to socio-demographic characteristics, MMSE and BDI scores (Table 1).

### Behavioral Data

The statistical analysis showed no significant difference between PD patients ON-levodopa (mean = 60%, SD = 17%) and OFF-levodopa (mean = 64%, SD = 25%; *t* = 0.519, *p* = 0.616) in overall accuracy. The overall performance of controls (mean = 80%, SD = 16%) was better than that of PD patients OFF-levodopa (*t* = 2.470, *p* = 0.024) and PD patients ON-levodopa (*t* = 2.408, *p* = 0.027). However in the AB condition, all the participants reached a high level of accuracy (controls: mean = 86%, SD = 13%; PD ON-levodopa: mean = 78%, SD = 21%; PD OFF-levodopa: mean = 73%, SD = 22%).

#### Testing Phase—Within Patients Analysis

**Figure 1:**
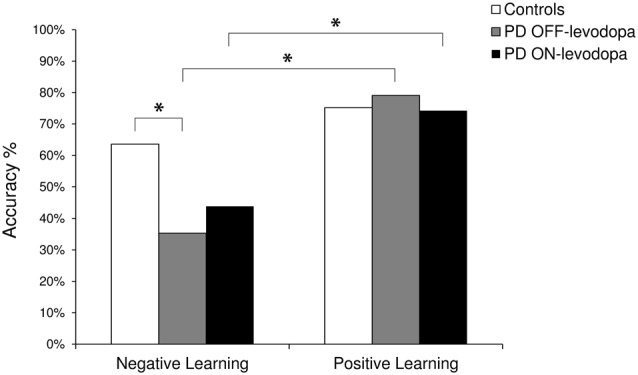
**The plot shows the percentage (%) of correct responses in the Testing Phase.** Negative Learning = percentage of avoidance of B responses; Positive Learning = percentage of choice of A responses. PD, Parkinson’s disease. *Indicates significant difference (*p* < 0.05).

Paired Samples *T* tests did not show any significant difference between PD patients ON-levodopa and PD patients OFF-levodopa in overall accuracy. To evaluate positive vs. negative learning we calculated the percentage of responses where subjects chose the stimulus A and the percentage of times the subjects avoided the stimulus B. The repeated measures ANOVA showed a significant effect of “Learning” (*F*_(1,9)_ = 17.417, *p* = 0.002). Multiple comparisons (Bonferroni corrected) revealed that the performance of PD patients was less accurate in negative learning (OFF-levodopa: mean = 35%, SD = 26%; ON-levodopa: mean = 44%, SD = 22%) than positive learning (OFF-levodopa: mean = 79%, SD = 12%; ON-levodopa: mean = 74%, SD = 23%) both OFF-levodopa (*p* = 0.003) and ON-levodopa (*p* = 0.038; Figure 1).

#### Testing Phase—Between Subjects Analysis

Independent Sample *T* test did not show any significant difference between ON-levodopa (mean = 60%, SD = 10%) and OFF-levodopa PD patients (mean = 58%, SD = 9%) and healthy controls (mean = 69%, SD = 16%) in overall accuracy. The comparison between controls and PD patients OFF-levodopa revealed a significant effect of “Learning” (*F*_(1,18)_ = 18.784, *p* = 0.000) indicating a better performance in positive learning than negative learning, “Group” (*F*_(1,18)_ = 4.740, *p* = 0.043) indicating a better performance in healthy controls than PD OFF-levodopa. A significant “Learning × Group” interaction was also found (*F*_(1,18)_ = 4.656, *p* = 0.045). Multiple comparisons (Bonferroni corrected) showed that PD patients OFF-levodopa (mean = 35%, SD = 26%) were less accurate than controls (mean = 63%, SD = 23%) in negative learning (*p* = 0.047) and PD patients OFF-levodopa were worse in negative learning (mean = 35%, SD = 26%) than positive learning (mean = 79%, SD = 12%; *p* < 0.001). The comparison between controls and PD patients ON-levodopa revealed a significant effect of “Learning” (*F*_(1,18)_ = 10.050, *p* = 0.005) indicating a better performance in positive learning than negative learning (Figure 1).

### Event-Related Potential Data

#### Testing Phase—Within Subjects Analysis

**Figure 2:**
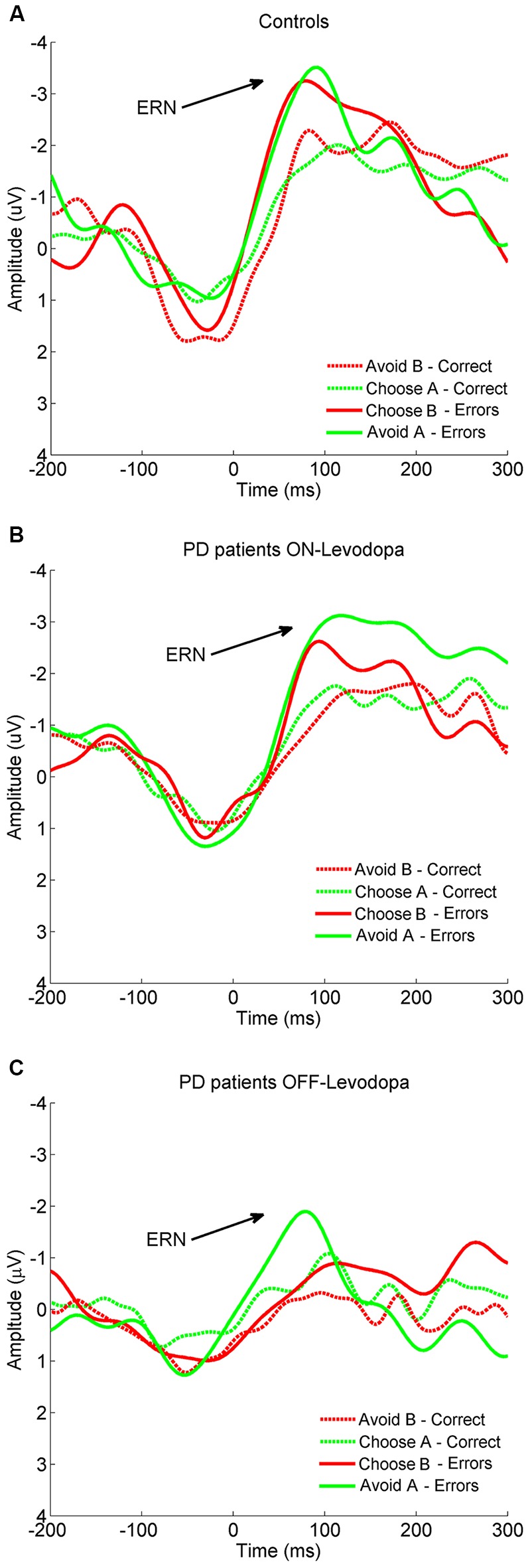
**Grand-average of response-locked event-related potentials (ERPs) to error and correct trials in (A) controls, (B) PD patients ON-levodopa and (C) PD patients OFF-levodopa at Cz.** PD, Parkinson’s disease.

Paired Samples *T* tests did not show any significant difference between PD patients ON-levodopa and PD patients OFF-levodopa in the overall ERN. Repeated measures ANOVA showed a significant effect of “Levodopa” (*F*_(1,9)_ = 24.830, *p* = 0.002) indicating a lower ERN amplitude in the OFF-levodopa state. Multiple comparisons (Bonferroni corrected) showed that PD patients OFF-levodopa (mean = 2.18 μV, SD = 1.33 μV) had a reduced ERN amplitude compared to ON-levodopa (mean = 3.95 μV, SD = 2.76 μV; *p* = 0.000) in their choice of B errors (*p* = 0.010; Figure 2).

#### Testing Phase—Between Subjects Analysis

Independent Sample *T* tests did not reveal any significant difference between PD patients ON-levodopa (mean = 2.71 μV, SD = 2.01 μV) and OFF-levodopa (mean = 2.10 μV, SD = 1.23 μV) and healthy controls (mean = 3.46 μV, SD = 1.83 μV) in overall amplitude. The comparison between healthy participants and PD patients OFF-levodopa (repeated measures ANOVA) showed a significant effect of “Group” (*F*_(1,18)_ = 5.193, *p* = 0.039) indicating a lower ERN amplitude in PD patients OFF-levodopa than controls. Multiple comparisons (Bonferroni corrected) showed that PD patients OFF-levodopa (mean = 2.18 μV, SD = 1.33 μV) had a significantly reduced ERN compared to controls (mean = 3.75 μV, SD = 2.36 μV) in their choice of B errors (*p* = 0.035). The comparison between healthy controls and PD patients ON-levodopa showed no significant effect (Figure 2).

## Discussion

In the behavioral results, we found no significant difference on overall accuracy between ON- and OFF-levodopa conditions. However, when we analyzed separately learning from positive vs. negative feedback, we found that performance of PD patients both ON- and OFF-levodopa was worse in learning from negative than positive feedback. Moreover, this difference was more pronounced in the OFF-levodopa condition, although it did not reach statistical significance. One possible explanation is that during the OFF-levodopa state, phasic dopamine dips associated to error detection are reduced because of the low level of levodopa. Moreover, dopamine agonists, in contrast to levodopa, tonically stimulate dopamine receptors preventing dips in dopamine transmission significantly impairing negative feedback learning (van Eimeren et al., 2009a). On the other hand, in the ON-levodopa state, a high level of levodopa enhanced phasic activity, improving learning both from positive and negative feedback (Schultz, 2002; Frank et al., 2004; Moustafa et al., 2013). It could be argued that the level of levodopa seems to have no modulatory effect on learning based on positive feedback since PD patients showed a performance similar to that of the controls both ON-levodopa and OFF-levodopa. However, OFF-levodopa PD patients were not completely washed out from levodopa medication and the minimum level of levodopa could have been high enough to ensure a sufficient phasic activity to sustain positive feedback learning.

The electrophysiological results support, at least in part, the behavioral data. The overall ERN amplitude did not differ among controls, ON-levodopa and OFF-levodopa PD patients. Analyzing separately ERN responses associated with the avoidance of the A stimulus (positive learning errors) vs. the choice of the B stimulus (negative learning errors), we found an effect of medication with the ERN amplitude associated to choice of B errors significantly reduced in the OFF-levodopa compared to the ON-levodopa state. Between-subjects analyses demonstrated also that the ERN amplitude associated with the choice of B errors was reduced in OFF-levodopa PD patients in comparison with controls. As for behavioral results, it could be assumed that the low level of levodopa negatively impacted on phasic activity rendering the ERN less sensitive to errors, and in particular to errors associated with negative learning. Moreover, persisting tonic stimulation of dopamine receptors—as with dopamine agonist medication—could therefore prevent dopamine dips and consequently reduce the ERN amplitude. On the other hand, when PD patients were in the ON-levodopa state, the high level of levodopa enabled phasic activity of mesencephalic dopamine neurons enhancing elicitation of the ERN.

In summary, we hypothesize that during the OFF-levodopa state, dopamine phasic activity (burst and dips) is reduced, causing an impairment in learning both from positive and negative feedback and a reduction of ERN amplitude to errors associated with both negative and positive learning. However, OFF-levodopa, the patients continue to experience the effect of dopamine agonists (via D2 receptors), determining a reduction of dopamine dips necessary for learning from negative feedback. Thus, in the OFF-levodopa state, learning from negative feedback and the ERN associated with negative errors are particularly affected. On the other hand, during ON-levodopa, both D1 and D2 receptors were activated. The activation of D1 receptors maintains dopamine phasic activity (both burst and dips) necessary for learning from feedback (both positive and negative), mitigating the effect of the dopamine agonists on the D2 receptors. However, these conclusions are weakened by the lack of a completely medication free group of PD patients. Since only the level of levodopa, but not the level of dopamine agonists has been manipulated, the hypothesis of a negative effect of dopamine agonists on learning from negative feedback and the ERN associated with choice of B errors needs to be confirmed by further studies in which both treatment with dopamine agonists and levodopa are manipulated to definitely discern the specific effect of one or the other substance.

The results obtained in this pilot study are in part in contrast with those previously reported by Frank et al. (2004, 2005). However, in our study, differently from Frank et al. (2004), patients were stable on dopamine agonist treatment. As mentioned above, impaired learning from negative feedback in the ON- and OFF-levodopa state could be, at least in part, attributable to dopamine agonists. Dopamine agonists, indeed, maintain a high tonic activity of dopamine neurons, preventing phasic response from being effective. A specific negative effect of dopamine agonists on punishment was previously reported in studies on learning in PD. Bódi et al. (2009) found that dopamine agonists positively affect reward processing whereas they impair punishment processing in never medicated PD patients. Cools et al. (2006) demonstrated that dopaminergic medication, and in particular the dopamine agonist pramipexole, impaired probabilistic and concurrent reversal learning in tasks where reversals were signaled by unexpected punishment in mild PD patients. According to the model of Frank (2005), phasic “dopamine dips” associated with error detection (ERN) are particularly vulnerable to the excessive dopamine levels following dopaminergic medication. When the dopamine level is too high because of the medication, “dopamine dips” are reduced leading to a selective impairment in learning from punishment. In line with these hypotheses, studies on impulse control disorder in PD have associated these behavioral defects with dopamine agonist treatment (Dodd et al., 2005; Antonini and Cilia, 2009; van Eimeren et al., 2009a; Cilia et al., 2011). Dopamine agonists contribute to the dysregulation of mesocortical dopaminergic pathways involved in the modulation of reward, positive vs. negative reinforcement learning, motivation, memory, inhibitory control and decision-making, and are thus closely implicated in impulse control and modulation of reward-seeking behaviors. Dopamine agonists, thus, could make PD patients less sensitive to negative reinforcement promoting errors based on a failure of learning from the negative consequences of their actions. Concordant with this clinical evidence Zalocusky et al. (2016) identified the role of the dopamine receptor type-2 in the nucleus accumbens in signaling unfavorable outcomes from the recent past at a time appropriate for influencing subsequent decisions. Moreover, they demonstrated that the systemic administration of the dopamine agonist pramipexole altered risk-preference in rats, increasing risk-seeking in a dose-dependent manner. In other words, dopamine agonists seem to alter the activity of D2 receptors in the nucleus accumbens disabling them to adequately signal negative outcomes. This assumption is also strengthened by functional imaging studies that examined the effect of dopamine agonists on neurofunctional activity related to reinforcement learning processes (van Eimeren et al., 2009a,b). Using functional magnetic resonance imaging, van Eimeren et al. (2009b) found that dopamine agonists (pramipexole) specifically changed activity of the orbitofrontal cortex in two ways that were both associated with increased risk taking in an out-of-magnet task. They propose that dopamine agonists prevent pauses in dopamine transmission and thereby impair the negative reinforcing effect of losing.

This result, if replicated, offers an interesting new possibility to interpret the differential effect of levodopa on behavior and cortical processing in PD: if the primary deficiency in the presence of a stable tonic dopamine level (or stable dopamine agonist level) consists of a reduced behavioral capacity for avoidance probably caused by impaired limbic- (anterior cingulate cortex) fronto-cortical processing of negative feedback reflected in the attenuated ERN, then psychopathological high risk behavior such as pathological gambling may be the consequence (van Eimeren et al., 2009a).

A possible limit of this study is that measuring the ERN during performance of the probabilistic learning task may seem inappropriate because of the uncertainty of response correctness. During the Learning Phase of the task the participants have formed a representation of what was an error or a correct response on the basis of probabilistic feedback. Hence, with different probability the same trial could be associated both to a positive and a negative feedback. It is likely that in the Testing Phase the participants judged some correct responses as errors, and vice versa, did not detect some errors. As demonstrated by previous studies (Pailing and Segalowitz, 2004; Frank et al., 2005), the ERN is elicited also when the response correctness is uncertain. In this condition, the ERN could be attenuated: if the ERN results from mismatches occurring during the response-comparison process, uncertainty could influence the outcome of the comparison process and, in some cases, the ERN mismatch signal would not occur, and the size of the ERN could be smaller in the averaged waveform (Pailing and Segalowitz, 2004). We believe that the question of uncertainty of response correctness is important but not critical for the interpretation of our findings. An ERN, indeed, was detectable in each group and condition and it was significantly reduced in PD patients OFF-levodopa during errors associated with negative learning. Regardless of the uncertainty of the response correctness, these results clearly indicate a selective effect to negative feedback only in PD patients OFF-levodopa.

Finally, because this is a pilot study with a small sample size, the results should be interpreted with caution, and further studies are needed to support our conclusions.

## Author Contributions

CV: analysis of data; drafting work; final approval of the version to be published; agreement to be accountable for all aspects of the work. SaS: conception of work; revising work critically; final approval of the version to be published; agreement to be accountable for all aspects of the work. SF: acquisition of data; drafting the work; final approval of the version to be published; agreement to be accountable for all aspects of the work. StS: analysis of data; revising work critically; final approval of the version to be published; agreement to be accountable for all aspects of the work. MC: acquisition of data; revising work critically; final approval of the version to be published; agreement to be accountable for all aspects of the work. FP, AA and NB: interpretation of data; revising work critically; final approval of the version to be published; agreement to be accountable for all aspects of the work.

## Funding

This study was supported by the Italian Ministry of Health, Current Research Funding (2590604), the Deutsche Forschungsgemeinschaft (DFG, Koselleck Project to NB), Brain Products Munich.

## Conflict of Interest Statement

The authors declare that the research was conducted in the absence of any commercial or financial relationships that could be construed as a potential conflict of interest. The reviewer JA and handling Editor declared their shared affiliation, and the handling Editor states that the process nevertheless met the standards of a fair and objective review.
